# Efficacy of Carbonate Buffer Mixture in Preventing Hoof Lamella Injury Associated with Subacute Ruminal Acidosis in Dairy Goats

**DOI:** 10.3390/vetsci11090395

**Published:** 2024-08-27

**Authors:** Maimaiti Tuniyazi, Ruibo Tang, Xiaoyu Hu, Naisheng Zhang, Peng Shen

**Affiliations:** Department of Clinical Veterinary Medicine, College of Veterinary Medicine, Jilin University, Changchun 130062, China; mmttn18@mails.jlu.edu.cn (M.T.);

**Keywords:** dairy goat, SARA, rumen microbiota, carbonate buffer mixture

## Abstract

**Simple Summary:**

This study investigated the efficacy of a carbonate buffer mixture (CBM) for preventing hoof lamella injury associated with subacute ruminal acidosis (SARA) in dairy goats over a 17-week period. Twenty-four healthy dairy goats were randomly assigned to control, SARA, or CBM groups. The SARA and CBM groups were subjected to a high-grain feeding regimen to induce SARA, and the CBM group received a daily CBM supplement of 10 g. The CBM group maintained a more stable rumen pH, had lower levels of inflammatory markers, and had a slightly lower incidence of hoof lamellar injury than the SARA group did. These findings suggest that long-term CBM supplementation may mitigate SARA-associated hoof lamella injury in dairy goats by regulating the rumen environment and reducing the levels of harmful metabolites.

**Abstract:**

Subacute ruminal acidosis (SARA) is a prevalent metabolic disorder in highly productive dairy cows that results in serious issues, including hoof lamellar injuries. This study aimed to investigate the efficacy of a carbonate buffer mixture (CBM) in preventing hoof lamella injury in dairy goats, a species also susceptible to SARA due to similar feeding practices over a 17-week period. Twenty-four healthy dairy goats were randomly assigned to three groups: control, SARA, and CBM groups. The control group received a standardized diet, whereas the SARA and CBM groups were subjected to a high-grain feeding regimen to induce SARA. The CBM group received a daily supplement of 10 g CBM mixed with their diet. Clinical assessments, including body temperature, rumen pH, inflammatory markers, matrix metalloproteinases (MMPs), and hoof lamellar injuries, were monitored throughout the study. The results showed that the CBM group maintained a more stable rumen pH and had lower levels of inflammatory markers than the SARA group did. The incidence of hoof lamellar injury was slightly lower in the CBM group. These findings suggest that long-term CBM supplementation may mitigate SARA-associated hoof lamella injury in dairy goats by regulating the rumen environment, fostering the growth of healthy bacterial communities, and by reducing the production of harmful metabolites. The use of CBM as a dietary supplement may have significant implications in improving the health, welfare, and productivity of dairy animals.

## 1. Introduction

Subacute ruminal acidosis (SARA) is a prevalent metabolic disorder that affects ruminants [1], especially high-yield dairy cows, at an alarming rate. This is largely due to the widespread use of high-grain diets by farmers to increase milk production, which can lead to various metabolic issues including SARA [2]. The disorder not only reduces milk yield and fat content in dairy cows, but also triggers several health problems, such as diarrhea, mastitis, and lamellar injury, resulting in considerable economic losses in the dairy farming industry [3,4]. This challenge has significant implications for herd productivity and welfare in the modern dairy industry.

SARA arises from dysregulation of ruminal pH, which leads to metabolic disturbances that affect microbial populations within the rumen and disrupt nutrient metabolism and absorption. The pH range defining SARA is typically between 5.5 and 6.0 [5,6]. Although SARA may not always present with overt clinical signs, it can have detrimental effects on the health, productivity, and welfare of the animals. One of the most significant consequences of SARA in dairy cows is the damage it causes to the hoof lamellae, which can lead to lameness, reduced feed intake, and impaired mobility. This ultimately compromises the overall performance and profitability of dairy cow operations.

The relationship between SARA and hoof lamella injury in dairy cows has been extensively documented in the scientific literature [7,8]. The underlying mechanism of this relationship involves the disruption of the rumen microbiota. Guo et al. discovered that the abundance of acid-producing bacteria in the rumen and the concentrations of lactic acid and LPS in the serum increased in cows with naturally occurring chronic lamellar injuries [9]. In contrast, Li et al. suggested that oligofructose overload leads to an increase in the relative abundance of *Lactobacillus* and *Streptococcus*, resulting in lactate accumulation and lower ruminal pH in sheep during acute lamella injury [10]. In both chronic and acute conditions, rumen-derived endotoxins and inflammatory mediators can initiate a cascade of events, ultimately leading to the breakdown of laminar structures within the hoof. Although dietary management strategies and the use of probiotics have shown some promise in mitigating the effects of SARA by regulating the rumen microbiota [11], their efficacy in preventing hoof lamella injury is lacking. In addition to rumen microbiota, studies have shown the presence of matrix metalloproteinases (MMPs) in the lamellar tissue of sheep [10], particularly MMP-1, MMP-2, and MMP-9 [12,13,14,15]. Although dysbiosis of the rumen microbiota may result in increased MMPs in both blood serum and lamella tissues, it remains unclear whether restoring rumen microbiota can decrease MMP levels, thereby alleviating lamella injury.

Several studies have demonstrated the favorable impact of carbonate buffer mixture (CBM) on animal health and productivity, primarily because of its ability to effectively decrease lactic acid and biogenic amine concentrations [16,17,18]. However, CBM has attracted our interest because of its significant role in maintaining optimal pH levels in the rumen environment. Consequently, CBM may have the potential to regulate the rumen environment, foster the growth of healthy bacterial communities, and reduce the risk of SARA by maintaining a more stable rumen pH and limiting the production of harmful metabolites, thereby alleviating SARA-associated lamellar injuries. Preventing SARA-related hoof lamellar injuries is essential to the overall health and well-being of dairy cattle. 

The primary objective of the present study was to evaluate the efficacy of CBM in preventing hoof lamella injury associated with SARA in dairy goats for more than 17 weeks. This study assessed the long-term effects of CBM supplementation on the rumen microbiota, rumen pH, inflammatory markers, MMPs, and the incidence of hoof lamellar injuries.

## 2. Materials and Methods

### 2.1. Animals and Diet Management

A total of 24 healthy dairy Saanen goats, aged 4 ± 0.75 years old and weighing 30 ± 3.6 kg, with an average number of two lactations and an average BCS of 3 [19], were included in this study. The goats were obtained from a dairy farm and raised under consistent and controlled conditions from birth for milk supply purposes. Goats were selected based on a thorough evaluation of their medical records, ensuring that they had no previous digestive disorders, recent exposure to antibiotics or anthelmintic treatments, or long-distance transportation within the past three months. The sample size was determined by power analysis using G*Power software (version 3.1.9.6; Heinrich-Heine-Universität Düsseldorf, Düsseldorf, Germany) [20,21]. With an expected effect size of 0.5, α = 0.1, and power (1 − β) = 0.88, a sample size of 8 goats per group was calculated.

All selected goats were tagged with numbers and randomly assigned to three different groups (control, SARA, and CBM) using self-developed Python-generated computer code (https://github.com/murdoch103/animal-sample-random.git), accessed on 22 September 2023.

To establish a highly controlled feeding environment, the selected goats were isolated and provided with a standardized diet that met their nutrient requirements (Table 1). Following the adaptation period, the control group continued to receive the same diet, whereas the SARA and CBM groups were subjected to a high-grain feeding regimen. To prevent any possible cross-contamination, the animals were maintained in different closed, temperature-controlled spaces (25 °C, humidity: 30%). All goats were fed daily at 08:00 and 17:00, had unlimited access to water, and did not receive any additional dietary supplements throughout the experiment.

The study was conducted at the University Animal Experiment Base, Lvyuan District, Jilin, China. The geographical location is between 43° E and 125° E. The average annual temperature is 20.1 °C. 

### 2.2. Induction of SARA

**Figure 1:**
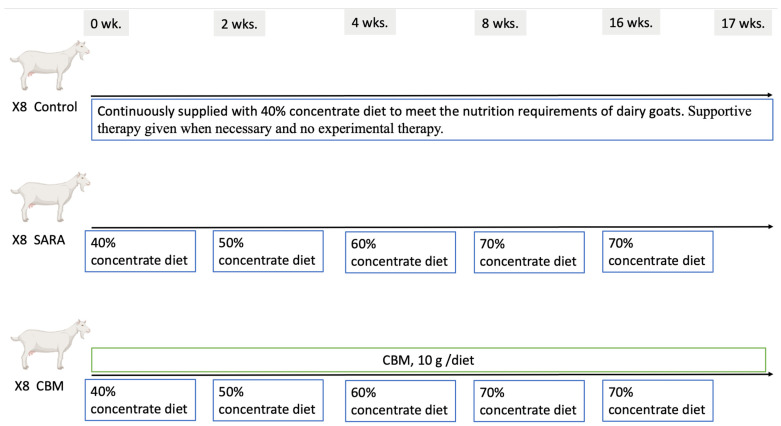
Study timeline overview.

Building on prior research [4,22], the current study experimentally induced SARA in dairy goats. To ensure proper adaptation, goats were acclimated to their assigned diets and housing conditions. Following this initial period, the concentrate diet was gradually increased over time for each goat in the experimental groups (SARA and CBM) (Figure 1). The concentration of the diets was slowly increased from 40% to 70% over 17 weeks, with an increase of 20% every two weeks.

### 2.3. Carbonate Buffer Mixture (CBM) Preparation and Administration

CBM concentration was determined before the start of this study. We added 5, 10, 20, and 30 g of CBM to a 40% concentration diet for four goats. After observing the leftover feed, we determined that 10 g CBM per diet was the best option. 

The CBM formulations used in this study are listed in Table 2. For administration to each goat in the CBM group, the chemical compounds were fully mixed in a bowl and 10 g of CBM was added to the diet of each goat. The mixture was freshly prepared immediately prior to each feeding session.

### 2.4. Clinical Assessments

Throughout the study, clinical assessments were conducted to evaluate various parameters related to the health and condition of the dairy goats. These assessments included body temperature, rumen fluid pH, body weight, and diarrhea score. Body temperature was measured using a rectal thermometer. The pH of the rumen fluid, which indicates acidity levels, was assessed using a pH meter on samples collected through a gastric tube. Diarrhea scoring in goats was performed using a previously established scale [23] with scores ranging from 0 to 5. A score of 0 represented normal feces, characterized by firm but moist balls that retained their shape. Scores increased as the consistency of the feces became softer, spreading out upon reaching the ground and eventually transforming into watery manure lacking any formed or recognizable pieces.

### 2.5. Serum Assessments

LPS Concentration Detection

Blood samples were collected and centrifuged at 14,000× *g* for 30 min at 4 °C. After centrifugation, the supernatants were transferred to sterile dehydrogenated glass tubes. The concentration of lipopolysaccharide (LPS) was determined using a chromogenic endpoint assay according to the manufacturer’s guidelines (MLBIO Biotechnology Co., Ltd., Shanghai, China). This assay had a minimum detection threshold of 0.01 EU/mL, allowing for precise measurements of LPS concentration.

Lactate Acid, Histamine, IL-10, IL-17A, MMP-1, MMP-2, and MMP-9 concentrations detection 

Blood samples were collected from different groups of horses and centrifuged at 3000× *g* for 30 min at 4 °C. Serum was collected and analyzed using an assay. Serum was collected and assayed for lactate, histamine, MMP-1, MMP-2, and MMP-9 levels using a commercial kit (MLBIO Biotechnology Co. Ltd.).

Total protein concentration detection

Blood samples were collected from goats in different groups and centrifuged at 3000× *g* for 30 min at 4 °C, and the resulting serum was collected for further analysis. The total protein concentration was determined using an IDEXX VetAutoread (IDEXX, Palo Alto, CA, USA). 

### 2.6. Euthanasia

Humane euthanasia was performed on the goats for evaluation of the lamellar pathology. The euthanasia protocol involved the use of a 1:5 (0.1 mL/kg) xylazine–ketamine combination (Lanzhou Fengsheng Pharmaceutical Co., Ltd., Lanzhou, China). The pre-euthanasia drug was injected intravenously through the jugular vein at a rate of 0.5–1 mL/sec. Sodium pentobarbital (Heilongjiang Feilong Pharmaceutical Co., Ltd., Suihua, China) was injected into the jugular vein at 0.1 mg/kg. To ensure the effectiveness of euthanasia, a corneal reflex test was conducted, in addition to a pinprick test on the ear surface, to confirm the absence of pain or consciousness. Observations of the absence of a heartbeat were also performed. The entire process was documented, and the protocol adhered to the ethical guidelines for animal research, with the oversight of a veterinary anesthetist and ethical approval from the relevant institutional review board.

Hematoxylin and eosin (H&E) and periodic acid–Schiff (PAS) staining. For pathological assessment of hoof tissues, all goats were humanely euthanized at the end of the experiment. Lamellar tissues were promptly collected within one hour of euthanasia and sectioned using a bandsaw. To obtain consistent samples, a 10 × 10 × 0.5 mm section was precisely cut from each hoof, encompassing both the hoof wall and lamellar tissue. These sections were subsequently divided into 55 mm square blocks, which were fixed in 4% formalin for 24 to 72 h. Fixation duration was consistent across all specimens, ensuring optimal tissue preservation. Following routine methods, the fixed blocks of lamellar tissue were embedded in paraffin wax. Particular attention was paid to maintain uniformity by consistently obtaining blocks from the same location within the lamellar tissue of each horse. To assess lamellar lesions and perform morphometric analysis, sections were stained using the established H&E and PAS staining methods based on the protocol described in previous studies [24]. Following staining, the sections were meticulously examined under a light microscope. To capture clear digital images, image capture software and a microscope-attached camera (Olympus, Tokyo, Japan) were utilized.

### 2.7. Microbial Assessments

Rumen fluid samples were collected from a cohort of 24 goats, with eight goats from each group, for bacterial DNA extraction and subsequent microbial analysis. DNA extraction was performed using the CTAB method following the manufacturer’s guidelines. This well-established method is effective for recovering DNA from minimal sample amounts, and has been validated for DNA preparation across diverse bacterial species. Blank samples were treated with nuclear-free water and served as negative controls. The extracted total DNA was eluted in 50 μL of elution buffer and stored at −80 °C for further analysis. To amplify the V3-V4 region of the 16S rDNA genes, a primer set 314F (5′-CCTACGGGNGGCWGCAG-3′) and 805R (5′-GACTACHVGGGTATCTAATCC-3′) was used, with barcodes attached to the 5′ ends of the primers to enable sample identification. Primers were designed to accommodate universal sequencing primers. PCR amplification reactions were performed using 25 ng of template DNA, PCR Premix, the respective primers, and PCR-grade water. The amplification conditions involved denaturation, annealing, and extension. The amplification products were verified by agarose gel electrophoresis. Negative controls with ultrapure water were included to rule out false positive PCR results. The PCR products were purified using AMPure XT beads and quantified using a Qubit. Subsequently, amplicon libraries were prepared, and their size and quantity were assessed using the Agilent 2100 Bioanalyzer and Library Quantification Kit for Illumina, respectively. Libraries were sequenced using the NovaSeq PE250 platform. The Illumina NovaSeq platform was used to sequence the samples, according to the manufacturer’s recommendations. Paired-end reads were associated with their respective samples using unique barcodes, and the primer sequences were removed. FLASH software (FLASH-1.1.11) was used to merge paired-end reads, and quality filtering was applied using fqtrim to generate high-quality clean tags. Chimeric sequences were removed using Vsearch (2.28.1) software after dereplication via DADA2 to create feature tables and sequences. To ensure consistency, the sequences were subjected to random normalization for alpha and beta diversity analyses. The SILVA database was employed for feature abundance normalization based on relative abundance. Alpha diversity indices (Chao1, Ace, Shannon, and Simpson) were used to assess the species diversity within the samples. Principal Coordinate Analysis (PCoA) was used to analyze microbial structure across different groups, and the identification of differentially abundant bacterial taxa among groups was performed using Linear Discriminant Analysis Effect Size (LEfSe) [25]. 

## 3. Statistical Analysis

All statistical analyses were performed using GraphPad Prism software (version 10.0.3 (217), GraphPad Software, Inc., San Diego, CA, USA). Data are presented as mean ± standard error of the mean (SEM). Statistical differences between the means were assessed using appropriate tests, including one-way analysis of variance (ANOVA), two-way ANOVA, and two-tailed *t*-tests. The alpha diversity of the rumen microbiota was calculated using the Kruskal–Wallis test, as applicable. Statistical significance was set at *p* < 0.05. Statistical significance was denoted by * *p* < 0.05, ** *p* < 0.01, *** *p* < 0.001, and **** *p* < 0.0001.

## 4. Results

**Figure 2:**
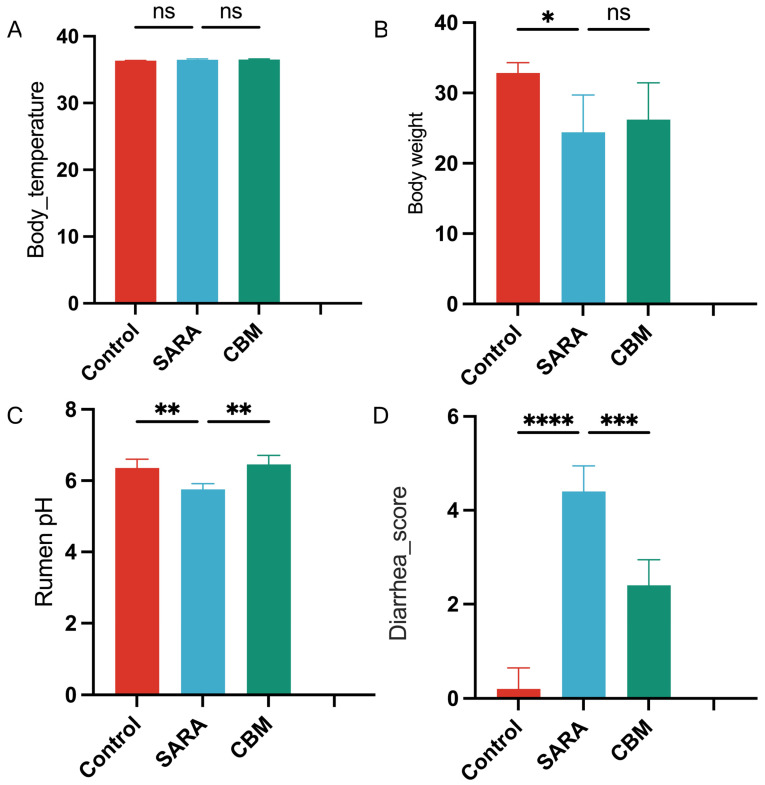
Clinical parameters of goats in different groups. (**A**) body temperature; (**B**) body weight; (**C**) rumen pH; and (**D**) diarrhea score. * *p* < 0.05, ** *p* < 0.01, *** *p* < 0.001, **** *p* < 0.0001, and ns not significant.

In our study, we evaluated the clinical attributes of goats across three distinct groups, including body temperature, body weight, rumen pH, and diarrhea score (Figure 2). 

In the SARA group, goats showed no significant changes in body temperature compared with the control group (*p* = 0.8011). In the SARA group, goats showed a significant decrease in body weight (*p* = 0.0201) and rumen pH (*p* = 0.0031) and a significant increase in diarrhea score (*p* < 0.0001) compared to the SARA group. In the CBM group, no significant changes were observed in body temperature (*p* = 0.916) or body weight (*p* = 0.6616), whereas a significant increase was observed in rumen pH (*p* = 0.0026) and a significant decrease in diarrhea score (*p* < 0.0001) compared to the SARA group. 

**Figure 3:**
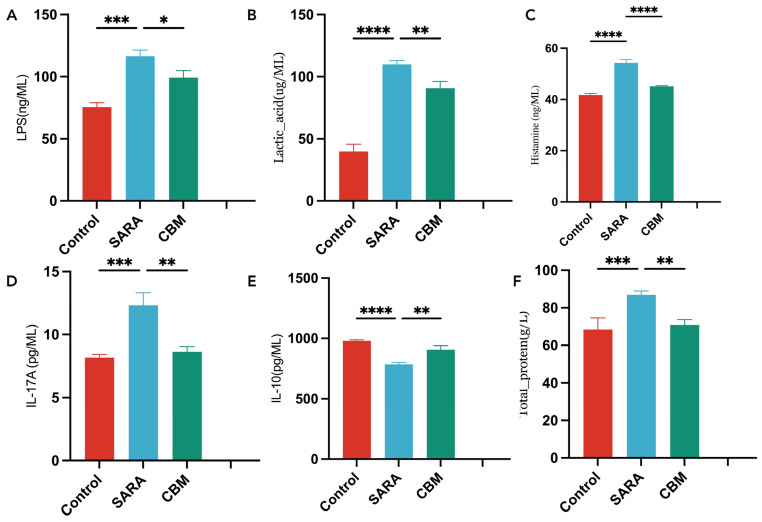
Blood serum concentrations of inflammatory cytokines in different groups. (**A**) LPS; (**B**) lactic acid; (**C**) histamine; (**D**) IL-17A; (**E**) IL-10; and (**F**) total protein. * *p* < 0.05, ** *p* < 0.01, *** *p* < 0.001, and **** *p* < 0.0001.

**Figure 4:**
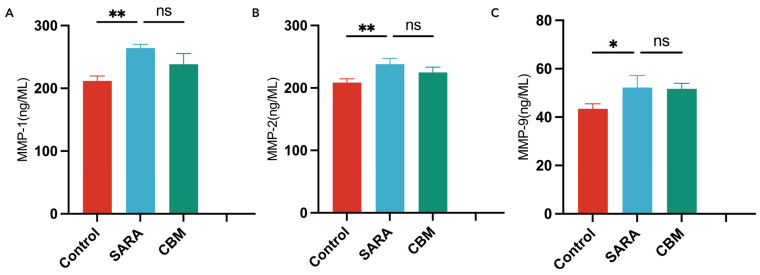
Blood serum concentrations of MMPs in different groups. (**A**) MMP-1; (**B**) MMP-2; and (**C**) MMP-9. * *p* < 0.05, ** *p* < 0.01, and ns not significant.

To evaluate the systemic inflammatory responses in our study subjects, we analyzed the blood serum levels among goats in distinct groups (Figure 3 and Figure 4). The results revealed significant differences, offering valuable insights into inflammatory markers. Specifically, goats in the SARA group showed significantly higher concentrations of LPS (*p* = 0.0005), lactic acid (*p* < 0.0001), histamine (*p* < 0.0001), IL-17A (*p* = 0.0003), total protein (*p* < 0.0001), MMP-1 (*p* < 0.0029), MMP-2 (*p* < 0.0031), and MMP-9 (*p* < 0.0301) than those in the control group but showed significantly lower concentrations of IL-10 (*p* < 0.0001). 

The CBM group showed a substantial reduction in the serum levels of LPS (*p* = 0.0412), lactic acid (*p* = 0.0092), histamine (*p* < 0.0001), IL-17A (*p* = 0.0097), and total protein (*p* = 0.0069), while showing a significantly increased concentration of IL-10 (*p* = 0.0083), compared to the SARA group. However, there were no significant changes in the concentrations of MMP-1 (*p* = 0.1545), MMP-2 (*p* = 0.2257), and MMP-9 (*p* = 0.3931) in the CBM group compared with those in the SARA group. 

Histopathology evaluation of lamellar tissues of goats in different groups

**Figure 5:**
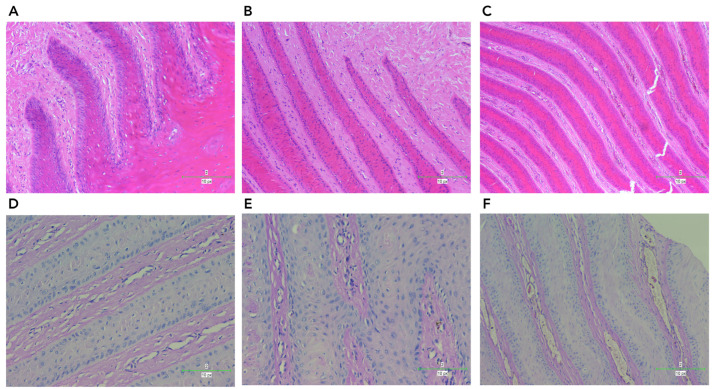
Histopathological observation of the hoof lamella tissues (×40) (H&E: (**A**–**C**); PAS: (**D**–**F**)). (**A**) control (H&E); (**B**) SARA (H&E); (**C**) CBM (H&E); (**D**) control (PAS); (**E**) SARA (PAS); (**F**) CBM (PAS).

To further investigate the changes in the lamellar tissue of dairy goats under CBM intervention, we conducted histopathological evaluations using H&E and PAS staining (Figure 5). Both staining methods clearly demonstrated the separation between the epidermal and dermal laminae in the hoof, a characteristic feature of lamellar injury, in the SARA group compared to the control group (Figure 5A,B,D,E). The SARA group also exhibited inflammatory cells, such as neutrophils and macrophages, as well as irregularities, distortion, and tissue rupture in the epidermal tissue (hoof wall). In the CBM group, supplementation improved the repair of damaged lamellar tissue structures (Figure 5C–F). Notably, the CBM group exhibited a more intact lamellar structure, similar to that observed in the control group. 

Evaluation of rumen microbiota profiles in different groups

Composition of the rumen microbiota in different groups

**Figure 6:**
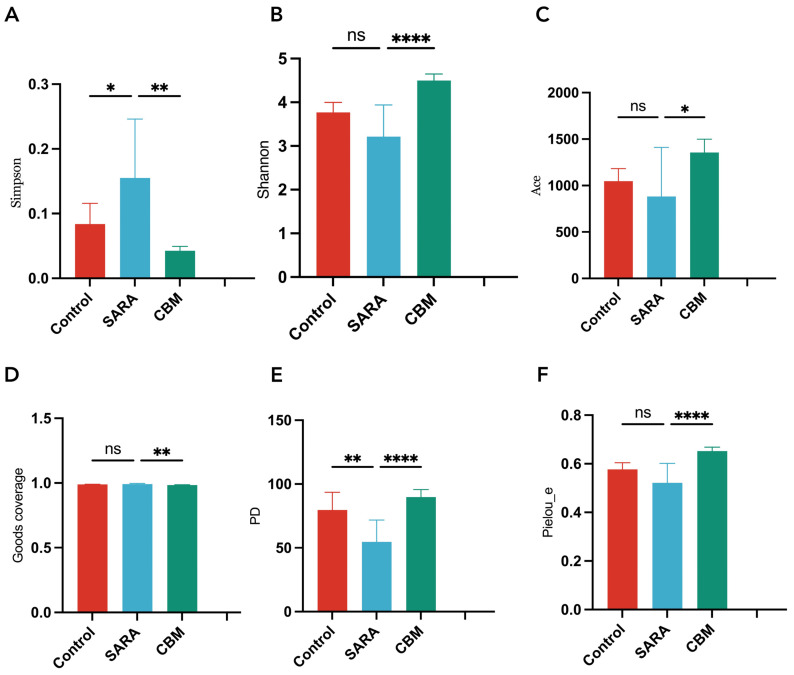
Simpson, Shannon, Ace, Goods_coverage, PD, and Pielou_e indices for different groups (**A**–**F**). * *p* < 0.05, ** *p* < 0.01, **** *p* < 0.0001, and ns not significant.

To assess bacterial diversity in our study, we generated rarefaction curves, which demonstrated that the sampling depth led to an ample number of sequences representing the majority of the bacterial diversity (Figure 6). We further examined bacterial richness and diversity, including Shannon, Simpson, Ace, Goods coverage, Pd, and Pielou-e indices. 

Our results showed that the Simpson index was significantly higher in the SARA group than in the control group (*p* = 0.0462). The Simpson index in the CBM group was significantly lower than that in the SARA group (*p* = 0.0017). The Shannon index, on the other hand, showed no significant change in the SARA group compared to the control group (*p* = 0.0532). However, the Shannon index of the CBM group was significantly higher than that of the SARA group (*p* < 0.0001). The ACE index did not show significant changes in the SARA group compared with the control group (*p* = 0.5737). The ACE index was significantly lower in the CBM group than in the SARA group (*p* = 0.0220). Good coverage showed no significant change in the SARA group compared to the control group (*p* = 0.2703). Good coverage was significantly higher in the CBM group than in the SARA group (*p* = 0.0022). Pd was significantly lower in the SARA group than in the control group (*p* = 0.0029). The Pd was significantly higher in the CBM group than in the SARA group (*p* < 0.0001). The Pielou_e index did not show significant changes in the SARA group compared with the control group (*p* = 0.0922). Pielou was significantly higher in the SARA group (*p* < 0.0001). 

**Figure 7:**
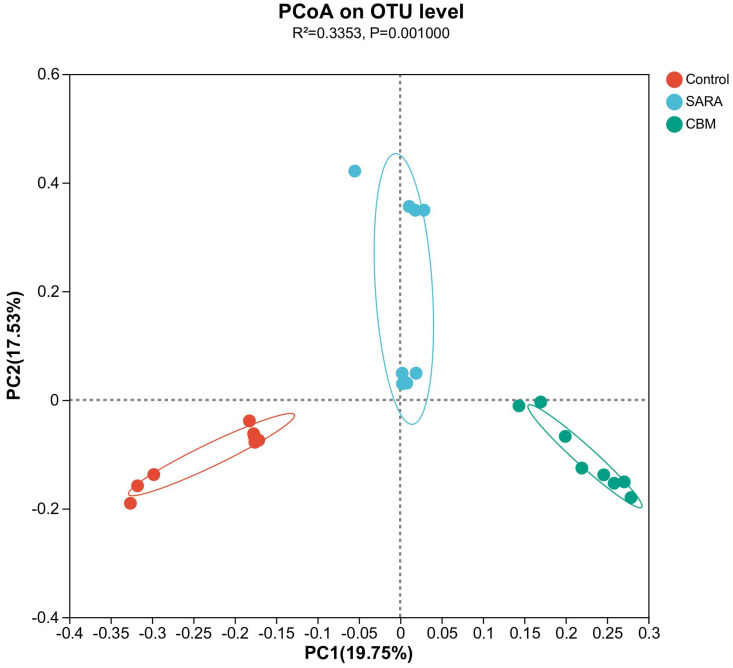
PCoA plot of rumen microbial composition in different groups.

Principal Coordinate Analysis (PCoA), based on the unweighted UniFrac distance, revealed the rumen bacterial architecture within distinct groups. The results showed distinct bacterial structures in the control, SARA, and CBM groups (*R2* = 0.3353, *p* = 0.001) (Figure 7).

Composition of the rumen microbiota at the Phylum level in different groups

**Figure 8:**
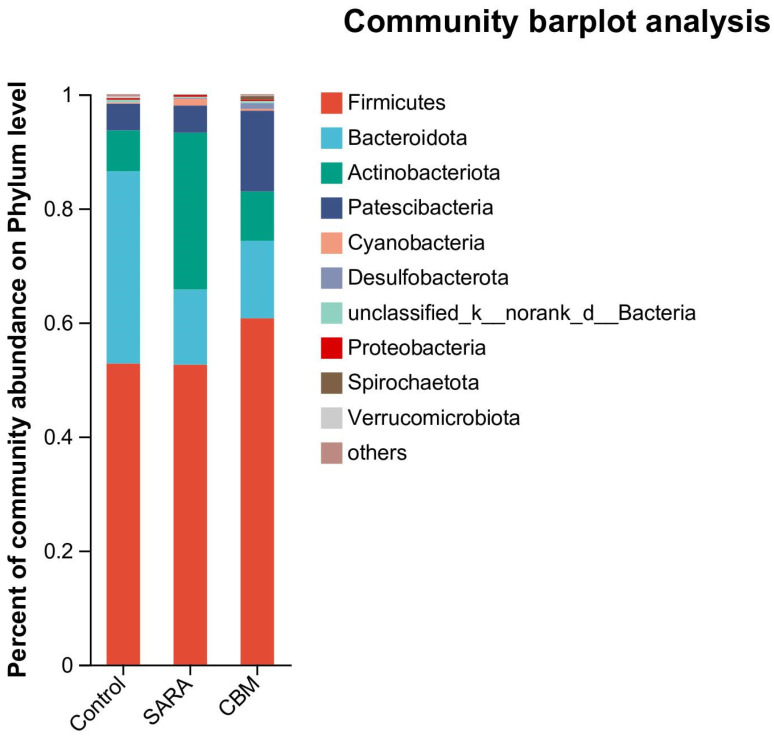
The rumen microbiota compositions in different groups at the phylum level.

To understand the composition of the rumen microbiota at the phylum level, we analyzed the top 10 phyla in goats from the different groups (Figure 8). 

In goats in the control group, the top 10 phyla were *Firmicutes* (54.25%), *Bacteroidota* (31.55%), *Actinobacteriota* (8.06%), *Actinobacteriota* (8.06%), *Patescibacteria* (4.35%), *unclassified_k__norank_d__Bacteria* (0.53%), Verrucomicrobiota (0.36%), *Proteobacteria* (0.32%), *Cyanobacteria* (0.24%), *Spirochaetota* (0.03%), *Desulfobacterota* (0.02%), and others (0.29%). The top ten phyla in the SARA group were *Firmicutes* (52.58%), *Actinobacteriota* (27.49%), *Bacteroidota* (13.18%), *Patescibacteria* (4.76%), *Cyanobacteria* (1.21%), *Proteobacteria* (0.27%), *unclassified_k__norank_d__Bacteria* (0.20%), *Desulfobacterota* (0.14%), *Spirochaetota* (0.10%), *Verrucomicrobiota* (0.04%), and others (0.04%). The top ten phyla in the CBM group were *Firmicutes* (60.71%), *Patescibacteria* (14.16%), *Bacteroidota* (13.61%), *Actinobacteriota* (8.63%), *Desulfobacterota* (1.02%), *Spirochaetota* (0.62%), *unclassified_k__norank_d__Bacteria* (0.38%), *Cyanobacteria* (0.33%), *Proteobacteria* (0.25%), *Synergistota* (0.19%), and others (0.12%).

Composition of the rumen microbiota at the Genus level in different groups

**Figure 9:**
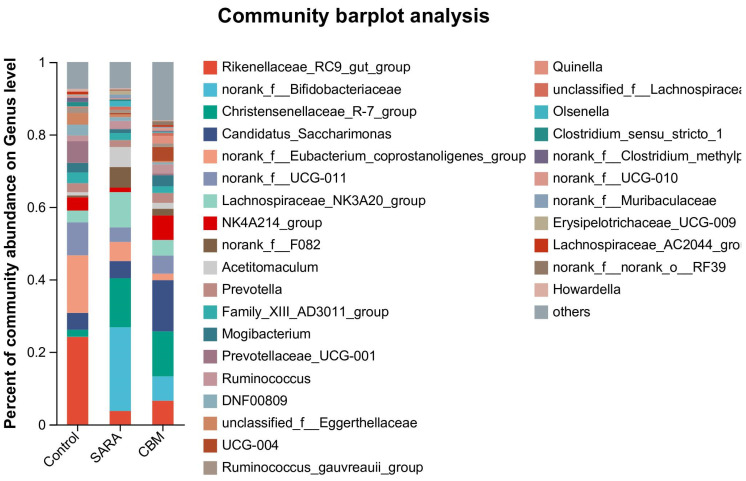
The rumen microbiota compositions in different groups at the genus level.

The top 10 genera at the genus level are shown in Figure 9. In goats in the control group, the top ten genera were *Rikenellaceae_RC9_gut_group* (24.12%), *norank_f__Eubacterium_coprostanoligenes_group* (15.89%), *norank_f__UCG-011* (9.07%), *Candidatus_Saccharimonas* (4.67%), *NK4A214_group* (3.65%), *Lachnospiraceae_NK3A20_group* (3.29%), *Christensenellaceae_R-7_group* (1.91%), *Acetitomaculum* (0.88%), *norank_f__F082* (0.52%), *norank_f__Bifidobacteriaceae* (0.06%), and others (35.94%). The top ten genera in the SARA group, the top 10 genera were *norank_f__Bifidobacteriaceae* (23.07%), *Christensenellaceae_R-7_group* (13.53%), *Lachnospiraceae_NK3A20_group* (9.77%), *norank_f__F082* (5.68%), *Acetitomaculum* (5.53%), *norank_f__Eubacterium_coprostanoligenes_group* (5.28%), *Candidatus_Saccharimonas* (4.75%), *norank_f__UCG-011* (3.98%), *Rikenellaceae_RC9_gut_group* (3.72%), *Ruminococcus* (2.11%), and others (22.58%). In goats in the CBM group, the top ten genera were *Candidatus_Saccharimonas* (14.14%), *Christensenellaceae_R-7_group* (12.38%), *NK4A214_group* (6.74%), *norank_f__Bifidobacteriaceae* (6.71%), *Rikenellaceae_RC9_gut_group* (6.55%), *norank_f__UCG-011* (5.00%), *Lachnospiraceae_NK3A20_group* (4.30%), *norank_f__F082* (1.86%), *norank_f__Eubacterium_coprostanoligenes_group* (1.79%), *Acetitomaculum* (1.63%), and others (38.87%).

**Figure 10:**
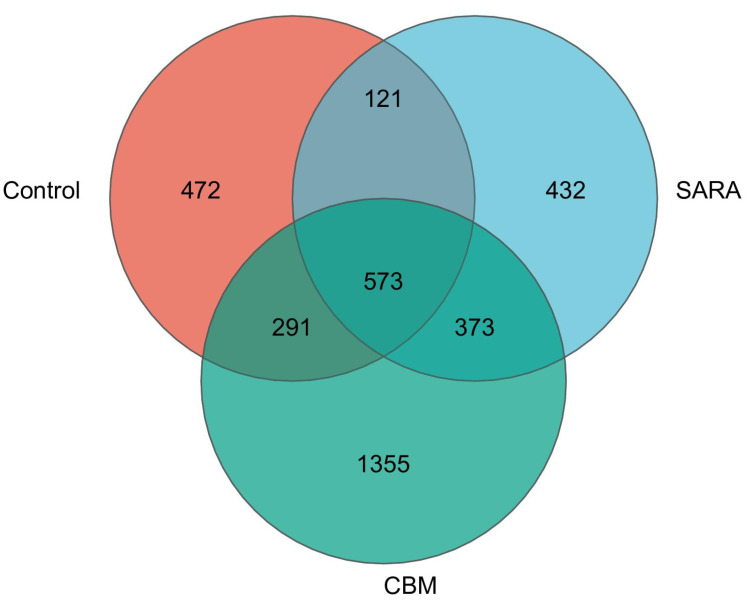
Venn diagram of shared core OUTs in different groups.

Moreover, we aimed to assess the connection between unique bacterial microbiota and SARA, as well as SARA and CBM supplementation, by examining the OUTs shared among goats from different groups. These findings revealed several core genera that were common among different groups of goats. The core genera accounted for only 39.33%, 38.23%, and 22.11% of the rumen microbiota in the control, SARA, and CBM groups, respectively (Figure 10). To better study the effects of core genera on development and prevention, we further analyzed the core genera. These results indicate that core genera may play an important role in the development of lamellar injuries in goats but have only limited effects on lamellar injury prevention.

**Figure 11:**
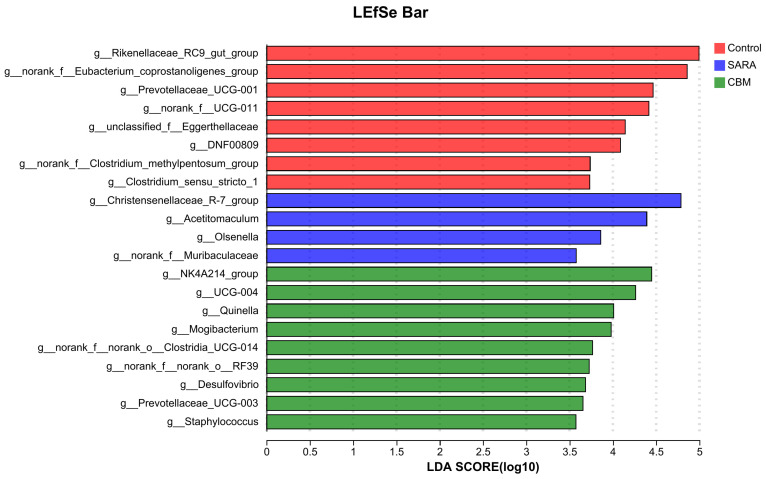
LEfSe analysis of the rumen microbiota compositions in different groups.

To conduct further investigation, we performed biomarker analysis using LEfSe, which involved the generation of an LEfSe plot (Figure 11). In the control group, at the genus levels, the biomarkers with significant discriminative power were *Rikenellaceae_RC9_gut_group*, *norank_f__Eubacterium_coprostanoligenes_group*, *Prevotellaceae_UCG-001*, *norank_f__UCG-011*, *unclassified_f__Eggerthellaceae, DNF00809*, *norank_f__Clostridium_methylpentosum_group*, and *Clostridium_sensu_stricto_1*. In the lamellar injury group, at the genus level, the biomarkers with significant discriminative power were *Christensenellaceae_R-7_group*, *Acetitomaculum g__Olsenella*, and *norank_f__Muribaculaceae.* At the genus level, in the CBM group, the biomarkers with significant discriminative power were *NK4A214_group*, *UCG-004*, *Quinella, Mogibacterium*, *norank_f__norank_o__Clostridia_UCG-014*, *norank_f__norank_o__RF39*, *Desulfovibrio*, *Prevotellaceae_UCG-003*, and *Staphylococcus*.

## 5. Discussion

This study investigated the prevention of high-grain SARA-related lamellar injury in dairy goats by manipulating the rumen microbiota. These results indicate that CBM supplementation effectively alleviated clinical signs, reduced inflammation, restored rumen microbiota balance, and prevented further hoof lamellae damage.

From a clinical standpoint, there were numerous distinctions between the goats in the control and SARA groups. Body weight, rumen pH, and diarrhea score were the most crucial factors. Body weight significantly reflects the performance and feeding efficacy of the animals. Our results showed that goats in the SARA group had notably lower body weights than those in the control group despite receiving a high-energy diet. These findings, along with previous studies linking SARA to decreased milk production and fat content [3,26], reduced dry matter intake [26], feed efficacy [26], and lameness [26], underscore the significant negative impact of SARA on animal production and welfare, particularly dairy and meat production. Thus, our findings suggest that a balanced diet with diverse nutritional contents may be more crucial than simply feeding animals with high-grain diets. The optimal diet may vary according to sex, age, breed, geographical location, season, and animal function, necessitating further research. Notably, CBM supplementation did not increase goat body weight compared to that in the SARA group, implying other regulatory mechanisms beyond the rumen microbiota or insufficient treatment duration. Future studies should investigate the effects of CBM without rumen microbiota and over extended treatment periods to understand the underlying mechanisms of body weight control.

Consistently, low rumen pH is a hallmark of SARA, which is prevalent in high-yield dairy cows within intensive production systems [27,28]. As previously noted [27], high-grain diets lower the rumen pH. Our study has limitations, as rumen pH is typically measured using rumen fluid collected noninvasively via a stomach tube, which can yield inaccurate readings if the tube contacts the mouth (pH 6.8–7.0) or esophagus (pH 5.5–6.5). Rumenocentesis, involving percutaneous needle aspiration from the caudoventral rumen, provides more precise pH data [29] but is less applicable in clinical settings because of owner concerns. Diagnosing SARA remains challenging due to the fluctuating nature of rumen pH, defined as consistently below 5.2–5.8 at any 3 h interval [30]. The acidic environment in the rumen disrupts the balance of microbiota and metabolites, leading to issues such as laminitis. CBM administration significantly increased rumen pH, which may mitigate such problems. The likely mechanism is CBM’s chemical composition of CBM, particularly CO3- and HCO3-, which combine with H+ ions to increase the pH levels. Additionally, CBM may alter the rumen environment, reduce acidity, and hinder lactic acid-producing bacteria, thereby decreasing acid accumulation and improving the pH levels. Increased pH levels may ultimately reduce hoof lamellar injuries.

Our study revealed a strong correlation between high levels of grain-induced SARA and an increased incidence of diarrhea, a common gastrointestinal disorder primarily caused by gut microbiota dysbiosis [31,32]. This result further indicates the involvement of rumen microbiota in lamellar injuries and suggests the rationality of manipulating the rumen microbiota to prevent lamellar damage. In addition, it is crucial to address diarrhea in animals. In the CBM group, the diarrhea score significantly decreased, indicating that CBM has potential therapeutic preventive effects on diarrhea symptoms and deserves further investigation.

Systemic inflammatory cytokines, including LPS, lactic acid, histamine, IL-17A, and total protein, were elevated in the SARA group compared with those in the control group, whereas the anti-inflammatory cytokine IL-10 was decreased. Conversely, the CBM group showed reduced levels of LPS, lactic acid, histamine, IL-17A, and total protein, and increased IL-10 levels compared to the SARA group.

LPS, a component of Gram-negative bacterial cell walls, significantly contributes to lamellar injury. Our study found increased serum LPS concentrations in the SARA group, indicating gastrointestinal barrier dysfunction and bacterial translocation, which perpetuates inflammation and intestinal barrier damage. Histamine, a biogenic amine associated with systemic inflammation, plays a crucial role in allergic and inflammatory responses [33]. We observed significantly elevated histamine concentrations in the SARA group, consistent with previous studies on equine and bovine lamellar injuries [34,35]. The current study observed a significant reduction in IL-10 concentration in the SARA group compared to that in the control group, indicating a specific inflammatory response pattern in the immune system [36,37]. IL-17A, a pro-inflammatory cytokine produced by Th17 cells [38], plays a critical role in inflammatory and autoimmune diseases and has been implicated in several conditions, including lamellar injury [39,40]. The IL-17A concentration was significantly higher in the SARA group than in the control group. Consistent with previous studies [41], we also found a significant increase in total protein concentration in the SARA group. This increase is likely due to inflammatory processes associated with altered rumen microbiota, leading to increased ruminal wall permeability and protein leakage into tissues, thus elevating total protein levels in the circulation. These findings highlight the importance of systemic inflammatory responses in SARA and lamellar injury development, suggesting that reducing inflammatory reactions could be a potential approach to maintaining hoof health in animals. Compared to the SARA group, the CBM group exhibited reduced levels of LPS, lactic acid, histamine, and IL-17A, along with increased IL-10 levels. These results suggest that CBM may improve the rumen microbiota balance, regulate metabolic processes, and reduce inflammation.

In the 1990s, researchers first noted altered enzymatic activity in the lamellar tissue. Later studies identified the activation of multiple MMPs (MMP-2 and MMP-9) in degrading extracellular matrix components within the lamellae [40]. These enzymatic changes lead to detachment of lamellar basal epithelial cells from the basement membrane, causing structural alterations typical of lamellar injury. MMP-1, or collagenase I, is secreted as a pro-enzyme and is activated by extracellular proteolytic removal of regulatory pro-peptides [42]. Our study found significantly elevated levels of MMP-1, MMP-2, and MMP-9 in goats with SARA-related lamellar injury compared with healthy goats, indicating the involvement of these enzymes in lamellar injury and their potential as therapeutic targets. These findings highlight the crucial role of MMPs in the pathogenesis of lamellar injury, and call for further research on their regulatory and therapeutic potential. However, MMP levels did not significantly change in the CBM group compared to those in the SARA group, suggesting that regulating the rumen microbiota alone may not reverse activated MMPs. Histological examination revealed subtle modifications, such as separation between the epidermal and dermal laminae [35], a hallmark of lamellar injury, in the SARA group compared to the control group. The lack of significant tissue damage improvement in the CBM group compared to the SARA group raises the question of CBM’s efficacy in reversing histological changes. These results suggest that more targeted treatments are required to address lamellar injuries. Therefore, reducing MMP levels may be a potential approach, and further investigation is warranted.

Our analysis of the rumen microbiota profiles provides a detailed view of the microbial dynamics associated with SARA and the potential impact of CBM therapy. The changes in alpha diversity metrics (Simpson, Shannon, Ace, Goods coverage, Pd, and Pielou_e) between the control, SARA, and CBM groups highlight the significant effects of ruminal acidosis on microbial richness and evenness. Distinct clustering in PCoA based on unweighted UniFrac distance indicated the resilience of rumen bacterial architecture to SARA and its potential modulation by CBM therapy.

At the phylum level, *Firmicutes*, *Bacteroidota*, and *Actinobacteriota* dominated across all groups. The altered abundance of these phyla in response to SARA and CBM therapy suggests that CBM has the potential to influence the rumen microbiota composition.

At the genus level, the dominant microbiota (>1%) in the control group were *Rikenellaceae_RC9_gut_group*, *norank_f__Eubacterium_coprostanoligenes_group*, *norank_f__UCG-011*, *Candidatus_Saccharimonas*, *NK4A214_group*, *Lachnospiraceae_NK3A20_group*, and *Christensenellaceae_R-7_group*. The dominant microbiota in the SARA group (>1%) included *norank_f__Bifidobacteriaceae*, Christensenellaceae_R-7_group, *Lachnospiraceae_NK3A20_group*, *norank_f__F082, Acetitomaculum*, *norank_f__Eubacterium_coprostanoligenes_group*, *Candidatus_Saccharimonas*, *norank_f__UCG-011*, *Rikenellaceae_RC9_gut_group*, and *Ruminococcus*. The dominant microbiota in the CBM group (>1%) were *Candidatus_Saccharimonas*, *Christensenellaceae_R-7_group*, *NK4A214_group*, *norank_f__Bifidobacteriaceae*, *Rikenellaceae_RC9_gut_group*, *norank_f__UCG-011*, *Lachnospiraceae_NK3A20_group*, *norank_f__F082*, n*orank_f__Eubacterium_coprostanoligenes_group*, and *Acetitomaculum*. The composition of rumen microbiota was similar among the different groups at the genus level. These findings suggest that CBM therapy can affect the composition of the rumen microbiota, as evidenced by the observed changes in the abundance of these phyla in response to both SARA and CBM supplementation. Biomarker analysis using LEfSe revealed specific genera associated with the control, SARA, and CBM groups. The identification of unique biomarkers underscores the potential of CBM to modulate specific bacterial taxa, thereby contributing to observed microbial shifts. These findings further indicate that rumen microbiota plays a crucial role in the development of SARA and lamellar injuries in rumenitis. However, the absence of significant improvement in the hoof tissues in the CBM group compared to the SARA group suggests that rumen microbiota manipulation may only have an assisting effect, and a lamella-targeted approach is needed. For example, MMP-inhibiting drugs include batimastat (BB-94) [43] and silymarin [44].

Currently, excessive selective pressure exerted by genetic selection companies has not only led to an increase in milk production but also to increased metabolic stress in the most productive subjects. This change has caused reproductive, health, and welfare issues as well as affects longevity. CBM supplementation may alleviate such problems in dairy animals fed a high-grain diet. Future studies should also consider assessing the efficacy of CBM in mitigating behavioral and productivity-related issues. In addition, educating future veterinarians, technicians, and farmers about the issues addressed in this paper and emphasizing the importance of a balanced diet as well as healthy rumen microbiota are crucial for enhancing animal welfare and productivity in the dairy and meat industry [45,46].

The above results suggest that CBM may have practical applications in both beef and dairy cattle and sheep and goat management, as most animal diseases are related to nutritional and metabolic disorders that result from rumen microbiota dysbiosis. High-grain diets are essential for providing sufficient energy for milk and meat production, and CBM supplementation may mitigate diet-related issues including SARA, laminitis, and behavioral issues. However, our study has certain limitations that warrant consideration. Notably, the absence of classic lamellar injury symptoms, such as reluctance to walk and extended periods of lying down, in goats with high-grain-induced SARA despite damage to the hoof lamella was notable. This difference may be attributed to the fact that goats are relatively smaller animals, and their hoof lamellar tissues are subjected to less compression than those of larger animals, such as horses and cows. Therefore, future studies should consider incorporating heavier animals to better examine the effects of CBM on lamellar injury. In addition, the lack of long-term follow-up in our study hindered our ability to assess the sustained clinical improvements or potential delayed side effects of CBM supplementation. To address this limitation, future studies should prolong the study period, carefully evaluate the long-term effects of CBM supplementation, and monitor for possible side effects that may arise over time. Further long-term studies should be conducted to provide clearer information and findings. Furthermore, the study primarily relied on rumen fluid collection via the stomach tube, which can yield inaccurate pH readings if the tube contacts the mouth or esophagus [47,48]. Although rumenocentesis offers more precise pH data, it is less practical in clinical settings. Therefore, future studies should consider the application of rumen cannula for both pH monitoring and sample collection. Finally, this study focused on the preventive effects of CBM on SARA-related lamellar injuries. However, the precise mechanisms by which CBM modulates the rumen microbiota and alters inflammatory responses remain to be fully elucidated. Future research should aim to decipher these mechanisms, potentially employing advanced techniques, such as whole-genome sequencing and metabolic profiling.

## 6. Conclusions

The findings of this study reveal that CBM administration can alleviate the incidence of hoof lamellar injuries associated with SARA in dairy goats. The preservation of a stable rumen pH and well-balanced rumen microbiota prevented an increase in the levels of inflammatory markers and detrimental metabolites, which are thought to contribute to the mitigating impact of CBM on SARA-related hoof lamellar injuries. However, its preventive effect was insufficient to prevent tissue damage, increase tissue repair, or decrease MMP levels. Although CBM supplementation showed promise in promoting overall animal health by regulating rumen microbiota and reducing inflammatory responses, additional research is necessary to explore a targeted approach for more effective prevention of SARA-associated hoof lamellar injuries in dairy animals.

## Figures and Tables

**Table 1:** Ingredient and nutritional composition of the experimental diets.

Items	Diets			
Ingredients composition, %DM	40%	50%	60%	70%
Alpha hay	56	44	36	26
Corn meal	32	40	48	56
Soybean meal	8	10	12	14
Limestone	2	2	2	2
Salt	1	1	1	1
Vitamin–mineral mix ^1^	1	1	1	1
Total	100.00	100.00	100.00	100.00
Nutrient composition				
Metabolic energy, MJ/kg DM	8.19	11.23	14.15	18.34
Crude protein, % DM	15	15	15	15
Crude fat, % DM	3.5	4	4	4
Neutral detergent fiber, % DM	52	25	25	25
Acid detergent fiber, % DM	35	15	15	15
Crude ash, % DM	10	5	5	5
Starch, % DM	17.12	23.13	29.87	37.95

^1^ Vitamin–mineral mix (per kilogram): 450 mg of nicotinic acid, 600 mg of Mn, 950 mg of Zn, 430 mg of Fe, 650 mg of Cu, 30 mg of Se, 45 mg of I, 20 mg of Co, 800 mg of vitamin E, 45,000 IU of vitamin D, and 120,000 IU of vitamin A.

**Table 2:** Chemical composition of CBM.

Name	Amount (g)	Percentage (%)
Na_2_CO_3_	7.5	8.5
NaHCO_3_	63	71.2
KCl	3	3.4
NaCl	15	16.9

## Data Availability

All data generated during the course of this study are included in the main article for reference and further analysis. Additionally, the 16S rRNA gene sequencing data from this study were deposited in the NCBI Sequence Read Archive (SRA) repository and can be accessed using the accession number PRJNA1118897.
